# Computer-Assisted Technique for Surgical Tooth Extraction

**DOI:** 10.1155/2016/7484159

**Published:** 2016-04-10

**Authors:** Hosamuddin Hamza

**Affiliations:** The Orthopaedic Department, October 6 University, Giza, Egypt

## Abstract

*Introduction*. Surgical tooth extraction is a common procedure in dentistry. However, numerous extraction cases show a high level of difficulty in practice. This difficulty is usually related to inadequate visualization, improper instrumentation, or other factors related to the targeted tooth (e.g., ankyloses or presence of bony undercut).* Methods*. In this work, the author presents a new technique for surgical tooth extraction based on 3D imaging, computer planning, and a new concept of computer-assisted manufacturing.* Results*. The outcome of this work is a surgical guide made by 3D printing of plastics and CNC of metals (hybrid outcome). In addition, the conventional surgical cutting tools (surgical burs) are modified with a number of stoppers adjusted to avoid any excessive drilling that could harm bone or other vital structures.* Conclusion*. The present outcome could provide a minimally invasive technique to overcome the routine complications facing dental surgeons in surgical extraction procedures.

## 1. Introduction

Surgical extraction of broken and badly decayed teeth is routinely done through conventional technique (root sectioning, bone cutting, and removal of dental and bony undercuts) [1]. This technique has proven fast and reliable; however, ideally, it needs 3 X-rays (periapical radiographs): 1 preoperative for evaluation of root curvature, angulation, or any root fracture; 1 intraoperative to check the accuracy of the extraction procedure (e.g., complete root sectioning); 1 postoperative to confirm complete removal of any remaining tooth structure [2].

This technique still possesses some limitations; for example, excessive bone cutting could lead to bone necrosis in the related area. Also, the technique is considerably invasive in areas related to vital structures (e.g., nerves and maxillary sinuses) when applied on teeth with fused or angled roots [3].

In addition, this conventional technique relies on visualization of the surgical field, which could be hindered in case of bleeding, irritable patients, overlapping soft tissues, or malaligned or malpositioned teeth. It also has a relatively prolonged operative time and could be not applicable in patients with limited mouth opening or incompliant patients [4].

While the use of 3D imaging, scanning, virtual designing, planning, and printing in dentistry has been increasing in the past decades, especially in dental implantation, the concept of printing of hybrid objects (metal and plastics) is limitedly applied in the medical and dental fields [5].

The aim of this work is to present a computer-assisted technique (cutting guide and instruments) that could replace the conventional surgical extraction.

## 2. Methods

For a patient undergoing surgical extraction for single or multiple teeth, a model is firstly done (impression and gypsum pouring) to validate the accuracy of the final outcome extraorally before proceeding with the surgical procedure. Then, the patient is directed to perform digital radiograph (CT or CBCT). The 3D images are firstly segmented so that teeth, bone, and other structures are differentiated.

Treatment planning is done according to 3D data collected from the radiographs, that is, accurate determination of the position, alignment, and inclination of teeth/roots. Also, bone density overall and in targeted areas could be determined from 3D radiographs.

Finally, vital structures such as nerves and sinuses adjacent to the proposed tooth/teeth for extraction are located.

The important aspects of the roots to be extracted are their position, angulation, and proximity to bone and other structures as well as their length and bifurcation/trifurcation depth [6].

The planning is done to provide cutting slots for the surgical burs to reach the roots, bone, and root-bone interface in optimal orientation. These cutting slots are designed to allow the drills to pass exactly and accurately in the weak areas between the roots as could be obtained from the 3D radiographs [7].

**Figure 1:**
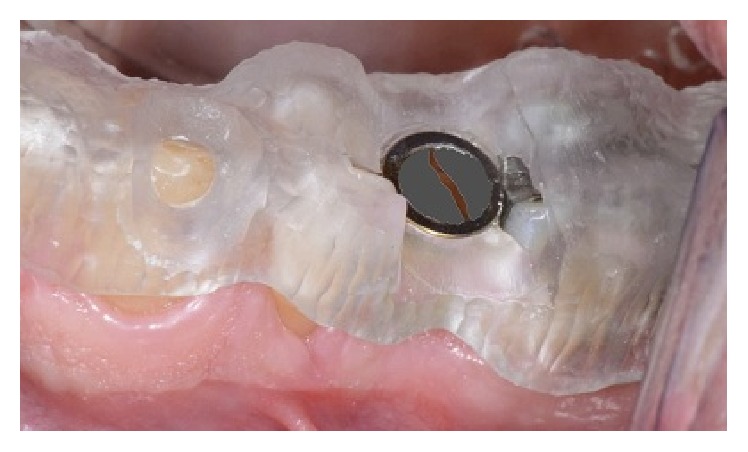
Surgical stent fabricated for extraction of remaining roots of mandibular first molar.

The weak areas are more likely to be in the junction between roots (Figure 1): in the lower molars, one cut in the line of junction between the mesial and distal roots, while in the upper molars, 3 cuts in the lines of junction between the mesiobuccal and distobuccal roots, between the mesiobuccal and palatal roots, and between the distobuccal and palatal roots.

Moreover, in case of existing bony undercut between one or more roots with the proximal bone, more cutting slots could be designed to make the drills pass accurately and undertake osteotomy to relieve any pressure in this area.

The cutting slots are designed as empty lines, points, or areas on the virtual stent corresponding to the cutting areas. The designing could be done by specifying the areas to be fabricated of metals and the rest of the stent to be fabricated of plastics. The surgical guide is designed with assuring the stability and extension of the stent and involvement of required areas as well as smoothening of its margins to avoid injury of the soft tissue due to friction.

Designing the plastic and metal components is done as two separate parts to account for internal structures, as well as include considerations for methods of fastening (jointing) the parts together into a single component.

Then, the final design of the surgical guide is converted to stl files, which are, in turn, transferred to special 3D printer that is capable of printing plastics, milling metal, and fusing metal and plastic material into one object.

The surgical guide is made of hybrid metal-plastic material. Each material is durable enough and heat-stable to withstand the mechanical forces during drilling and the heat during preoperative sterilization. The metal components are NiTi or stainless steel; while the plastics are polyamide nylon. The materials used do not result in any debris during drilling or cutting that could contaminate the surgical field and delay healing of the extraction socket.

**Figure 2:**
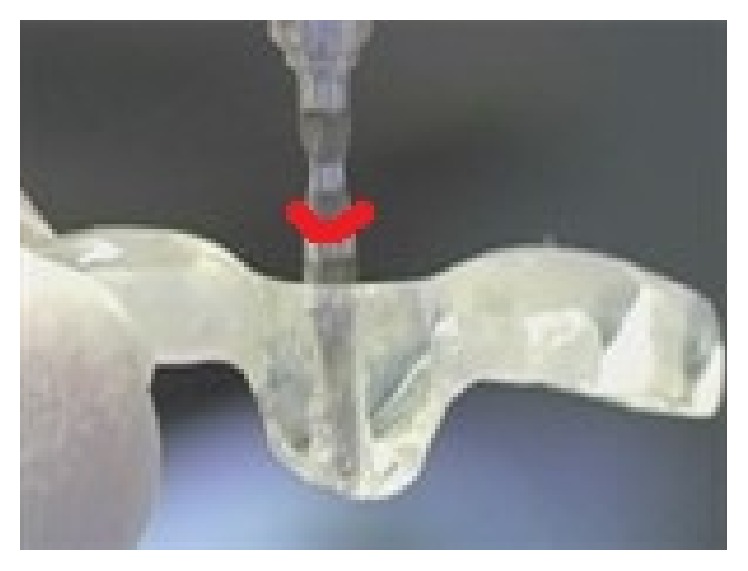
Hard stopper added to the cutting tool to determine the depth of cutting.

In another step of the technique, and according to measurements obtained from the 3D radiographs, hard plastic stoppers could be added to the drill to determine the depth of cutting (Figure 2) to avoid undesired overcutting (especially in depth dimension).

The surgical guide is adapted onto the master model to validate its fitting; then it could be sterilized preoperatively with autoclave. The cutting stent is fixed onto the patient's maxillary or mandibular arch by either pins, screws, or engagement into the dental or bone undercuts. Cutting could be done within the cutting slots by dental drills or burs with the depth stoppers. Cutting aims at freeing all possible undercuts around the targeted tooth that could resist the extraction procedure. Tooth extraction is completed safely by normal curved forceps or elevators.

## 3. Results

The surgical guide is specifically tailored for each patient with one or multiple teeth that need surgical extraction. The final outcome is a surgical guide that is fabricated to provide an access toward the surgical field. Visibility is not as important as the stent is capable of providing direct access to the target area. Surgical extraction using the surgical guide could minimize postoperative bleeding, tissue laceration, and pain.

## 4. Discussion

This work aims to exploit the currently evolving 4D printing technology which merges metallic and nonmetallic materials into one component or object. It also aims to ease surgical extraction procedure for dentists/oral surgeons and to reduce the pain that is usually associated with such procedure through planning the cutting direction, depth, and inclination on computer and then transferring these data into physical objects (templates and instrumentation) and to avoid uncalculated bone cuttings or teeth sectioning done in the procedures.

The proposed method is similar to fabricating the surgical guides routinely used for dental implants, as digital treatment plan is firstly performed with the data acquired from CT scan; a master model is created with extraction site determined prior to planning; segmentation of bone, teeth, and soft tissues is done on the digital scan.

Other geometric features are determined, including position and size of the target tooth for extraction as well as root angulation, inclination, and depth and the preferable drilling options [8]. Virtual evaluating of the treatment plan should be done prior to printing the splint and must be discussed with the surgeon performing the extraction procedure. However, modifying the treatment plan to simulate the tooth extractions or bone modifications should be done if necessary. Optionally, the surgical guide could be manufactured based on the master model; but, in the proposed method, the master model is used only for validating the final outcome [9].

Bone structure is segmented from CT image data, and the cutting slots on the guide are virtually added onto the soft tissue, bone, and tooth structures with parameters reflecting the treatment plan. The most important vital structure related to the maxillary jaw is the maxillary sinus, while, for the mandibular jaw, the nerve channels are of importance [10].

A model of the soft tissue can be further created from CT scan or optical scan to be united with or trimmed by jaw bone structure to give more detailed description of the surgical field. The thickness of soft tissue is digitally created and visually inspected; it should be equal to the distance between the cutting guide and the patient's jaw bone; calculation of soft tissue thickness is done by computing and analyzing this distance. The final outcome (surgical guide) has 2 surfaces: one fitting surface toward the patient's anatomy and an outside surface, which have to be checked against the master model before application in the surgery [11].

It is worth mentioning that advancing CAD/CAM and imaging technologies have enabled clinicians to analyze patients' anatomy and to manipulate areas that need skeletal reconstruction. It has been used for maxillofacial and implant procedures such as maxillary-sinus augmentation with reportedly high precision [12]. Moreover, in light of sinus augmentation procedures, the present method is similar to the guided bone-grafting and bone-reconstructive surgery which aim to reduce mental navigation and to replace the conventional surgical methods. The use of CT scanning and stereolithography has produced accurate and predictable results and enhanced the outcome of dental implant procedures [13].

One limitation of this work is that it relies on sophisticated 3D designing and needs special 3D printing and milling machines. However, depending on the components' shape considerations, each part of the surgical guide could be created with a different setup. When designing such components, some major differences between 3D printing (an additive process) and CNC milling (a subtractive process) should be considered. Additive processes build layer by layer and therefore have very little problem building internal structures. So, internal mesh, cavities, tubes, and the like inside a relatively seamless, solid piece (considering overhangs and support structures, here) could be created, but some formulations of plastics will not be available in 3D printer filaments coinciding with the metal part. The subtractive process, CNC milling, will provide more material flexibility, but it will require considering the shape of the components more carefully for the fabrication method. Soft metals like brass and aluminum could be used more accurately with relative ease, and could be milled through nylon easily. As milling is a subtractive procedure, it cuts from the outside of a material to the inside; that is, the procedure will be similar to cutting from a direction, rather than building layer by layer, so it could be difficult to get at the inside of the material without clearing away more material to have access to the internal portions of the components.

Another limitation of this methodology is the fact that it needs prolonged time compared to the routine surgical extraction done in dental facilities. However, considering the accurate cutting the surgical guide could provide and the less tissue injury due to trauma, tissue laceration, or uncalculated drilling, using this technique could compensate for the long time for achieving CT scans as well as designing and fabricating the cutting guide to complete the procedure [3].
